# P-th moment and almost sure stability of stochastic switched nonlinear systems

**DOI:** 10.1186/s40064-016-3686-z

**Published:** 2016-11-24

**Authors:** Haibo Gu, Caixia Gao

**Affiliations:** School of Mathematical Sciences, Inner Mongolia University, Hohhot, 010021 People’s Republic of China

**Keywords:** Stochastic switched nonlinear system, P-th moment stability, Almost sure stability, Multiple Lyapunov functions approach, Primary 60H10, 34D20; Secondary 93C30

## Abstract

This paper mainly tends to utilize $\psi$-type function to investigate p-th moment and almost sure stability for a class of stochastic switched nonlinear systems. Based on the multiple Lyapunov functions approach, some sufficient conditions are derived to check the stability criteria of stochastic switched nonlinear systems. One numerical example is provided to demonstrate the effectiveness of the proposed results.

## Background

Switched system is an indispensable class of hybrid dynamical systems, which is composed of a family of subsystems and a rule that orchestrates the switching among them. Yet, there exist stochastic effects in the practical systems such as physics, biology, information science and economic. (for instance, see Xie and Wang 2003; Zhang and Nie 2004; Goetz and Hritonenko 2008). Over the previous few decades, stochastic switched systems have received much attention due to their potential applications in many fields, such as the control of mechanical systems, automotive industry, aircraft and air traffic control, chemical and electrical engineering, etc., see e.g. Krystul (2001), Øksendal (2005).

As is well known, stability is one of the major issues in the study of control theory. In particular, the existence, uniqueness and stability of solutions for stochastic systems are investigated in Mao (1997), Hu et al. (2008), Zhang and Chen (2004) and the stability results of switched systems are given in Hu et al. (1999) and Aleksandrov et al. (2011). Some exponential stability, almost sure exponential stability and p-th moment stability criteria are obtained for stochastic systems in Mao (1994), Khasminskii (1980), Shen and Wang (2009). Recently, some efforts have been made to extend the stability results from stochastic systems to stochastic switched systems (Feng and Zhang 2006; Feng et al. 2011; Chatterjee and Liberzon 2004; Filipovic 2009; Ai and Zong 2014). In Branicky (1998), Dimarogonas and Kyriakopoulos (2004) and Chatterjee and Liberzon (2006) the investigators utilize multiple Lyapunov functions approach to study the stability of stochastic switched systems. P-th moment exponential stability of stochastic switched systems is investigated in Zhang et al. (2014) and Wu et al. (2013) and almost sure exponential stability of stochastic switched systems is researched in Cong et al. (2011). Although the stability of stochastic switched systems has stirred some initial research interest, there still leaves much room for further investigations to reduce the possible conservations.

For instance, in Hu et al. (2008), Wu and Hu (2012) and Pavlovic and Jankovic (2012), the researchers introduce $\psi$-type function and investigate p-th moment and almost surely $\psi ^{\gamma }$ stability for stochastic nonlinear systems. Since $\psi ^{\gamma }$ stability contains exponential stability and polynomial stability, it has a wide applicability. However, there are few research results about p-th moment and almost sure $\psi ^{\gamma }$ stability for stochastic switched nonlinear systems.

In this paper, we attempt to investigate p-th moment and almost sure $\psi ^{\gamma }$ stability of stochastic switched nonlinear systems. Since the switching behavior exists among stochastic switched systems, the stability of subsystems does not guarantee the stability of the whole system. By the aid of the semi-martingale convergence theorem, we obtain the p-th moment $\psi ^{\gamma }$ stability of stochastic switched nonlinear systems. In order to establish the criterion on almost surely $\psi ^{\gamma }$ stable of stochastic switched nonlinear systems, we improve the exponential martingale inequality in this paper.

The paper is organized as follows. Firstly, the problem formulations, definitions of $\psi ^{\gamma }$ stability and some lemmas are given in “Preliminaries” section. In third section, the main results on p-th moment $\psi ^{\gamma }$ stability and almost surely $\psi ^{\gamma }$ stability of stochastic switched nonlinear systems are obtained using multiple Lyapunov functions. An example is presented to illustrate the main results in “Examples” section . In the last section the conclusions are given.

## Preliminaries

Throughout this paper, unless otherwise specified, we let $R^{n}$ be the n-dimensional Euclidean space; $R_{+}$ is the set of all non-negative real numbers; $R^{n\times m}$ denotes the $n\times m$ real matrix space; $|\cdot |$ denotes the standard Euclidean norm for vectors; $\mathcal {C}^{1,2}(R_{+}\times R^{n})$ denotes the family of all non-negative functions *V*(*t*, *x*(*t*)) on $R_{+}\times R^{n}$ which are twice continuously differentiable in *x* and once in *t*; $L^{p}(\Omega ,R^{n})$ denotes the family of $R^{n}-$valued random variables $\xi$ with $E|\xi |^{p}<\infty$; $a \bigvee b$ denotes the maximum of a and b; $\mathcal {L}^{p}([a,b],R^{n})$ denotes the family of $R^{n}-$valued $\mathcal {F}_{t}-$adapted processes $\{f(t)\}_{a\le t\le b}$ such that $\int _{a}^{b}|f(t)|^{p}dt<\infty \ a.s.$; $P(\cdot )$ means the probability of a stochastic process; $E[\cdot ]$ means the expectation of a stochastic process; $\mathbb {N}={1,2,\ldots ,N}$ is a discrete index set, where *N* is a finite positive integer.

Consider a family of stochastic switched nonlinear systems described by

1 $$\begin{aligned} \left\{ \begin{aligned}&dx(t)=f_{\sigma (t)}(t,x(t))dt+g_{\sigma (t)}(t,x(t))dw(t) \\&x(t_{0})=x_{0},\quad t_{0}=0 \end{aligned} \right. \end{aligned}$$

where $\sigma (t): [t_{0},\infty )\rightarrow N$ is the switching signal, let $\{t_{1}<t_{2}<\cdots<t_{k}<\cdots \}$ be a switching sequence and the $i_{k}$-th subsystem is active at time interval $[t_{k},t_{k+1}]$, where $i_{k}$ is the switching instant, $i_{k}\in \mathcal {N}, \ k=0, 1, 2, \ldots$. System (1) is consisted with many stochastic subsystems $dx(t)=f_{i}(t,x(t))dt+g_{i}(t,x(t))dw(t)$ which are driven by switching signal $\sigma (t)$. $x(t)\in R^{n}$ is the state of the system, *w*(*t*) is an m-dimensional Brownian motion defined on the complete probability space $(\Omega ,\mathcal {F},\{\mathcal {F}_{t}\},P)$, with filtration $\mathcal {F}_{t}$ satisfying the usual conditions (i.e. it is increasing and right continuous while $\mathcal {F}_{0}$ contains all P-null sets), functions $f:R_{+}\times R^{n}\rightarrow R^{n}$, $g:R_{+}\times R^{n}\rightarrow R^{n\times m}$ are locally Lipschitz in $x(t)\in R^{n}$ and piecewise continuous in *t* for all $t\ge t_{0}$ and $f(t,0)=0, \ g(t,0)=0,t\in {[t_{0},\infty )}$.

For the existence and uniqueness of the solution we impose an assumption (A):

Both $f_{i}(t,x(t))$ and $g_{i}(t,x(t))$ satisfy the Lipschitz condition and the linear growth condition. That is, there exist a group of constants $L_{i}>0$ such that

$$\begin{aligned} |f_{i}(t,x)-f_{i}(t,y)|^{2}\vee |g_{i}(t,x)-g_{i}(t,y)|^{2}\le L_{i}\Vert x-y\Vert ^{2} \end{aligned}$$

For all $t\ge 0$, and $x,y\in R^{n}$, and, moreover, there is a group of constants $K_{i}>0$ such that

$$\begin{aligned} |f_{i}(t,x)|^{2}\vee |g_{i}(t,x)|^{2}\le K_{i}(1+\Vert x\Vert ^{2}) \end{aligned}$$

For all $t\ge 0$, and $x\in R^{n}$.

The purpose of this paper is to investigate the pth moment and almost sure $\psi ^{\gamma }$ stability of system (1), we first introduce some definitions as follows.

### **Definition 1**

(Hu et al. 2008) $\psi (t): R_{+}\rightarrow (0,1]$ is said to be $\psi$-type function, if it satisfies the following conditions:

1. It is continuous and differentiable;
2. $$\psi (0)=1$$ and $$\psi (t)\rightarrow 0$$, as $$t \rightarrow \infty$$;
3. $$\psi _{1}(t)=\psi '(t)/\psi (t)<0$$;
4. for any $$t,s\ge 0, \psi (t+s)\ge \psi (t)\psi (s)$$.

### **Definition 2**

For $p>0$, the stochastic switched nonlinear system (1) is said to be p-th moment $\psi ^{\gamma }$ stable, if there exist positive constants $\beta ,\gamma$ and function $\psi (\cdot )$ defined above, such that

2 $$\begin{aligned} E|x(t,x_{0})|^{p}\le \beta |x_{0}|^{p}\psi ^{\gamma }(t) \end{aligned}$$

when $p=2$, we say that it is $\psi ^{\gamma }$ stable in mean square, when $\psi (t)=e^{-t}$, we say that it is p-th moment exponential stable, when $\psi (t)=(1+t)^{-1}$,we say that it is p-th moment polynomial stable.

### **Definition 3**

Stochastic switched nonlinear systems (1) is said to be almost surely $\psi ^{\gamma }$ stable, if there exist a positive constant $\gamma$ and function $\psi (\cdot )$ defined above, such that

3 $$\limsup _{t\rightarrow \infty }\frac{\ln |x(t,x_{0})|}{|\ln \psi (t)|}\le -\gamma ,\quad a.s.$$

when $\psi (t)=e^{-t}$, we say that it is almost surely exponential stable, when $\psi (t)=(1+t)^{-1}$, we say that it is almost surely polynomial stable.

Before giving some efficient lemmas, let us introduce $It\hat{o}$ formula. For system (1), give any function $V(t,x)\in \mathcal {C}^{1,2}(R_{+}\times R^{n})$ and define operators *dV*(*t*, *x*) and $\mathcal {L}V(t,x)$ as follows.

$$\begin{aligned} dV(t,x)&= {} \mathcal {L}V(t,x)dt+V_{x}(t,x)g(t,x)dw(t) \\ \mathcal {L}V(t,x)&= {} V_{t}(t,x)+V_{x}(t,x)f(t,x)+\frac{1}{2}tr[g^{T}(t,x)V_{xx}(t,x)g(t,x)] \end{aligned}$$

where

$$\begin{aligned} V_{t}(t,x)=\frac{\partial V(t,x)}{\partial t}, V_{x}(t,x)=\left( \frac{\partial V(t,x)}{\partial x_{1}}, \ldots , \frac{\partial V(t,x)}{\partial x_{n}}\right) ,V_{xx}(t,x)=\left( \frac{\partial ^{2}V(t,x)}{\partial x_{i}\partial x_{j}}\right) _{n\times n} \end{aligned}$$

### **Lemma 4**

(Hu et al. 2008; Semi-martingale Convergence Theorem) *Let M*(*t*) *is a real value continuous local martingale, and* $M(0)=0, a.s.$, $\zeta$ *is an* $\mathcal {F}_{0}$ *measurable non-negative random variable, if* *X*(*t*) *is an* $\mathcal {F}_{t}$ *adapted continuous non-negative process and satisfies with*

$$\begin{aligned} X(t)\le \zeta + M(t),\quad t\ge 0 \end{aligned}$$

*then*, $EX(t)\le E\zeta$, *and* *X*(*t*) *is bounded* *a*.*s*., *that is*

$$\begin{aligned} \limsup _{t\rightarrow \infty }X(t)<\infty ,\ a.s. \end{aligned}$$

### **Lemma 5**

(Mao 1997; Doob’s Martingale Inequalities) *Let* $\{M_{t}\}_{t\ge 0}$ *be an* $R^{n}$-*valued martingale. Let* [*a*, *b*] *be a bounded interval in* $R_{+}$.

1. if $p\ge 1$ and $M_{t}\in L^{p}(\Omega ,R^{n})$, then for all $c>0$,
  $$\begin{aligned} P(\sup _{a\le t\le b}|M_{t}(\omega )|\ge c)\le c^{-p}E|M_{b}|^{p}. \end{aligned}$$
2. if $p> 1$ and $M_{t}\in L^{p}(\Omega ,R^{n})$, then
  $$\begin{aligned} E(\sup _{a\le t\le b}|M_{t}|^{p})\le \left( \frac{p}{p-1}\right) ^{p}E|M_{b}|^{p}. \end{aligned}$$

In order to establish the criterion on almost sure $\psi ^{\gamma }$ stability of stochastic switched nonlinear systems, we need to modify the exponential martingale inequality as follows.

### **Lemma 6**

(Exponential Martingale inequality) *Let* $g\in \mathcal {L}^{2}([0,T]\times R^{n})$, $\forall \varepsilon ,\delta >0$, *then*

4 $$\begin{aligned} P\left( \sup _{0\le t\le T}\left[ \int _{0}^{t}\frac{|\ln \psi (t)|}{t}g(s)dw(s)-\frac{\delta }{2}\int _{0}^{t}\frac{\ln ^{2} \psi (t)}{t^{2}}|g(s)|^{2}ds\right] \ge \varepsilon \right) \le e^{-\delta \varepsilon }. \end{aligned}$$

### *Proof*

For any positive integer $n\ge 1$, define a stopping time:

$$\begin{aligned} \tau _{n}=\inf \left\{ t\ge 0 : |\int _{0}^{t}\frac{|\ln \psi (t)|}{t}g(s)dw(s)|+\int _{0}^{t}\frac{\ln ^{2} \psi (t)}{t^{2}}|g(s)|^{2}ds>n \right\} \end{aligned}$$

and a process:

$$\begin{aligned} y(t)=\delta \int _{0}^{t}I_{[0,\tau _{n}]}(s)\frac{|\ln \psi (t)|}{t}g(s)dw(s)-\frac{\delta ^{2}}{2}\int _{0}^{t}I_{[0,\tau _{n}]}(s)\frac{\ln ^{2} \psi (t)}{t^{2}}|g(s)|^{2}ds, \end{aligned}$$

where $[0,\tau _{n}]$ is a stochastic time interval. When the set

$$\begin{aligned} \left\{ t\ge 0 : |\int _{0}^{t}\frac{|\ln \psi (t)|}{t}g(s)dw(s)|+\int _{0}^{t}\frac{\ln ^{2} \psi (t)}{t^{2}}|g(s)|^{2}ds>n \right\} \end{aligned}$$

is empty, $\tau _{n}=\inf \phi =\infty$, obviously, $\tau _{n}\uparrow \infty$. By $It\hat{o}$ formula, we have

$$\begin{aligned} \begin{aligned} e^{y(t)}=&1+\int _{0}^{t}e^{y(s)}\left[\delta I_{[0,\tau _{n}]}(s)\frac{|\ln \psi (t)|}{t}g(s)dw(s)-\frac{\delta ^{2}}{2}I_{[0,\tau _{n}]}(s)\frac{\ln ^{2} \psi (t)}{t^{2}}|g(s)|^{2}ds\right] \\&+\frac{1}{2}\delta ^{2}\int _{0}^{t}I_{[0,\tau _{n}]}(s)\frac{\ln ^{2} \psi (t)}{t^{2}}|g(s)|^{2}ds \\ =&1+\delta \int _{0}^{t}e^{y(s)}I_{[0,\tau _{n}]}(s)\frac{|\ln \psi (t)|}{t}g(s)dw(s) \end{aligned} \end{aligned}$$

That is, $e^{y(t)}$ is a non-negative martingale for all $t\ge 0$, $E[e^{y(t)}]=1$.

By the first part of Lemma 5 with $p=1$, we obtain

$$P\left(\sup _{0\le t\le T}y(t)\ge \delta \varepsilon \right)=P\left(\sup _{0\le t\le T}e^{y(t)}\ge e^{\delta \varepsilon }\right)\le e^{-\delta \varepsilon }E[e^{y(T)}] =e^{-\delta \varepsilon }.$$

That is

$$\begin{aligned} \begin{aligned} P&\left( \sup _{0\le t\le T}\left[ \int _{0}^{t}I_{[0,\tau _{n}]}(s)\frac{|\ln \psi (t)|}{t}g(s)dw(s)-\frac{\delta }{2}\int _{0}^{t}I_{[0,\tau _{n}]}(s)\frac{\ln ^{2} \psi (t)}{t^{2}}|g(s)|^{2}ds\right] \ge \varepsilon \right) \le e^{-\delta \varepsilon }. \end{aligned} \end{aligned}$$

Let, $n\rightarrow \infty$, we have

$$\begin{aligned} P\left( \sup _{0\le t\le T}\left[ \int _{0}^{t}\frac{|\ln \psi (t)|}{t}g(s)dw(s)-\frac{\delta }{2}\int _{0}^{t}\frac{\ln ^{2} \psi (t)}{t^{2}}|g(s)|^{2}ds\right] \ge \varepsilon \right) \le e^{-\delta \varepsilon }. \end{aligned}$$

So, the proof is completed. $\square$

### **Lemma 7**

(Mao 1997; Borel-Cantelli’s Lemma) *If* $\{A_{k}\}\subset \mathcal {F}$ *and* $\sum _{k=1}^{\infty }P(A_{k})<\infty$, *then*

$$\begin{aligned} P(\limsup _{k\rightarrow \infty }A_{k})=0. \end{aligned}$$

*That is, there exist a set* $\Omega _{o}\in \mathcal {F}$ *with* $P(\Omega _{o})=1$ *and an integer valued random variable* $k_{o}$ *such that for every* $\omega \in \Omega _{o}$ *we have* $\omega \notin A_{k}$ *whenever* $k\ge k_{o}(\omega )$.

## Main results

In this section, we shall tend to investigate a family of stochastic switched nonlinear systems by using multiple Lyapunov functions approach and give some sufficient conditions estimating the p-th moment and almost surely $\psi ^{\gamma }$ stable. Before giving the efficient theorems, we assume that the switching signal $\sigma (t)$ is right continuous.

Let us turn our attention to system (1) and give some sufficient results.

### **Theorem 1**

*For stochastic switched nonlinear systems* (1), *let (A) hold, if there exist a group of Lyapunov functions* $V_{i}(t,x)\in \mathcal {C}^{1,2}(R_{+} \times R^{n})$ *and positive constants* $p,b_{i},c_{i},\gamma$, *and* $\eta \ge 1$, *such that, for all* $t\ge 0,\ x\in R^{n}$

5 $$b_{i}|x|^{p}\le {} V_{i}(t,x)\le c_{i}|x|^{p}$$

6 $$\mathcal {L}V_{i}(t,x)\le {} \gamma \psi _{1}(t)V_{i}(t,x)$$

*and at each switching instant* $t_{k},\ (k=1,2,\ldots )$,

7 $$\begin{aligned} V_{\sigma (t_{k})}(t_{k},x(t_{k}))\le \eta V_{\sigma (t_{k-1})}(t_{k},x(t_{k})) \end{aligned}$$

*Then, for every* $x_{0}\in R^{n}$, *there exists a solution* $x(t)=x(t,x_{0})$ on $[t_{0},\infty )$ *to stochastic switched nonlinear system* (1). *Moreover, the system* (1) *is p-th moment* $\psi ^{\gamma }$ *stable and*

8 $$\begin{aligned} E|x(t,x_{0})|^{p}\le \frac{c}{b}\Vert x_{0}\Vert ^{p}\psi ^{\gamma }(t). \end{aligned}$$

### *Proof*

Let $x(t)=x(t,x_{0})$ is a solution of stochastic switched nonlinear system (1) and $h(t)=\psi ^{-\gamma }(t)E|x(t,x_{0})|^{p}$.

We can give switching signal $$\sigma (t)$$ and instant *t* for arbitrary, and assume that $$t_{k}$$ is the last switching instant before *t*, i.e. there is no switching on the interval $$[t_{k},t)$$.

By condition (5), we obtain

$$\begin{aligned} \begin{aligned} b_{\sigma (t_{k})}\psi ^{-\gamma }(t)|x(t,x_{0})|^{p} & \le\psi ^{-\gamma }(t)V_{\sigma (t_{k})}(t,x(t)) \\ &=\psi ^{-\gamma }(t_{k})V_{\sigma (t_{k})}(t_{k},x(t_{k}))+\int _{t_{k}}^{t}d[\psi ^{-\gamma }(s)V_{\sigma (t_{k})}(s,x(s))] \\ &=\psi ^{-\gamma }(t_{k})V_{\sigma (t_{k})}(t_{k},x(t_{k}))+\int _{t_{k}}^{t}\psi ^{-\gamma }(s)[-\gamma \psi _{1}(s) V_{\sigma (t_{k})}(s,x(s))+\mathcal {L}V_{\sigma (t_{k})}(s,x(s))]ds \\&+ \int _{t_{k}}^{t}\psi ^{-\gamma }(s)\frac{\partial V_{\sigma (t_{k})}(s,x(s))}{\partial x}g_{\sigma (t_{k})}(s,x(s))dw(s) \\ & \le\eta \psi ^{-\gamma }(t_{k-1})V_{\sigma (t_{k-1})}(t_{k},x(t_{k}))+\int _{t_{k}}^{t}\psi ^{-\gamma }(s)[-\gamma \psi _{1}(s) V_{\sigma (t_{k})}(s,x(s))+\mathcal {L}V_{\sigma (t_{k})}(s,x(s))]ds \\&+\int _{t_{k}}^{t}\psi ^{-\gamma }(s)\frac{\partial V_{\sigma (t_{k})}(s,x(s))}{\partial x}g_{\sigma (t_{k})}(s,x(s))dw(s) \\ & =\eta \psi ^{-\gamma }(t_{k-1})V_{\sigma (t_{k-1})}(t_{k-1},x(t_{k-1}))+\int _{t_{k-1}}^{t}\psi ^{-\gamma }(s)[-\gamma \psi _{1}(s) V_{\sigma (s)}(s,x(s))+\mathcal {L}V_{\sigma (s)}(s,x(s))]ds\\&+\int _{t_{k-1}}^{t}\psi ^{-\gamma }(s)\frac{\partial V_{\sigma (s)}(s,x(s))}{\partial x}g_{\sigma (s)}(s,x(s))dw(s)\\ & \le\eta ^{k}\psi ^{-\gamma }(t_{0})V_{\sigma (t_{0})}(t_{0},x(t_{0}))+\int _{t_{0}}^{t}\psi ^{-\gamma }(s)[-\gamma \psi _{1}(s) V_{\sigma (s)}(s,x(s))+\mathcal {L}V_{\sigma (s)}(s,x(s))]ds \\&+\int _{t_{0}}^{t}\psi ^{-\gamma }(s)\frac{\partial V_{\sigma (s)}(s,x(s))}{\partial x}g_{\sigma (s)}(s,x(s))dw(s) \end{aligned} \end{aligned}$$

Let $t_{0}=0$, we obtain

$$\begin{aligned} \begin{aligned} b_{\sigma (t_{k})}\psi ^{-\gamma }(t)|x(t,x_{0})|^{p}&\le\eta ^{k} V_{\sigma (t_{0})}(0,x_{0})+\int _{0}^{t}\psi ^{-\gamma }(s)\left[-\gamma \psi _{1}(s) V_{\sigma (s)}(s,x(s))+\mathcal {L}V_{\sigma (s)}(s,x(s))\right]ds\\&+\int _{0}^{t}\psi ^{-\gamma }(s)\frac{\partial V_{\sigma (s)}(s,x(s))}{\partial x}g_{\sigma (s)}(s,x(s))dw(s) \end{aligned} \end{aligned}$$

By condition (5), we have

9 $$\begin{aligned} \begin{aligned} b_{\sigma (t_{k})}\psi ^{-\gamma }(t)|x(t,x_{0})|^{p} & \le\eta ^{k} c_{\sigma (t_{k})}|x_{0}|^{p}+\int _{0}^{t}\psi ^{-\gamma }(s)[-\gamma \psi _{1}(s)V_{\sigma (s)}(s,x(s)) +\mathcal {L}V\sigma (s)(s,x(s))]ds+M(t) \end{aligned} \end{aligned}$$

where $M(t)=\int _{0}^{t}\psi ^{-\gamma }(s)\frac{\partial V_{\sigma (s)}(s,x(s))}{\partial x}g(s,x(s))dw(s)$ is a continuous local martingale with initial value $M(0)=0$.

Substituting (6) into (9) results in

$$\begin{aligned} b_{\sigma (t_{k})}\psi ^{-\gamma }(t)|x(t,x_{0})|^{p} \le \eta ^{k} c_{\sigma (t_{k})}|x_{0}|^{p}+M(t). \end{aligned}$$

By Lemma 4, we have $b_{\sigma (t_{k})}h(t)\le \eta ^{k} c_{\sigma (t_{k})}|x_{0}|^{p}$. Then

$$\begin{aligned} E|x(t,x_{0})|^{p}\le \frac{c_{\sigma (t_{k})}\eta ^{k}}{b_{\sigma (t_{k})}}\Vert x_{0}\Vert ^{p}\psi ^{\gamma }(t). \end{aligned}$$

Let $b=\min \{b_{i}\{$ and $c=\max \{c_{i}\eta ^{k}\}$, we obtain

$$\begin{aligned} E|x(t,x_{0})|^{p}\le \frac{c}{b}\Vert x_{0}\Vert ^{p}\psi ^{\gamma }(t), \quad t\ge 0. \end{aligned}$$

Thus, the system (1) is p-th moment $\psi ^{\gamma }$ stable. $\square$

### *Remark 1*

Here we generalize our research to stochastic switched nonlinear systems. The result shows how to derive some useful conditions for stochastic switched systems in terms of the multiple Lyapunov functions method.

In the following, the almost sure $\psi ^{\gamma }$ stability of system (1) is presented.

### **Theorem 2**

*For stochastic switched nonlinear system* (1), *let (A) hold, if there exist positive constants* $p,b_{i},\gamma , \eta \ge 1$, *and a group of Lyapunov functions* $V_{i}(t,x)\in \mathcal {C}^{1,2}(R_{+}\times R^{n})$, *such that, for all* $t\ge 0$ *and* $x\ne 0$

10 $$\begin{aligned} b_{i}|x|^{p}\le & {} V_{i}(t,x) \end{aligned}$$

11 $$\begin{aligned} \mathcal {L}V_{i}(t,x)\le & {} \gamma \psi _{1}(t)V_{i}(t,x) \end{aligned}$$

*and at each switching instant* $t_{k},\ (k=1,2,\ldots )$,

12 $$\begin{aligned} V_{\sigma (t_{k})}(t_{k},x(t_{k}))\le \eta V_{\sigma (t_{k-1})}(t_{k},x(t_{k})) \end{aligned}$$

*Then, for every* $x_{0}\in R^{n}$, *there exists a solution* $x(t)=x(t,x_{0})$ *on* $[t_{0},\infty )$ *to stochastic switched nonlinear system* (1). *Moreover, the system* (1) *is almost surely* $\psi ^{\gamma }$ *stable and*

13 $$\begin{aligned} \limsup _{t\rightarrow \infty }\frac{\ln |x(t,x_{0})|}{|\ln \psi (t)|}\le -\frac{\gamma }{p} \quad a.s.. \end{aligned}$$

### *Proof*

Clearly, (13) holds for $x_{0}=0$ since $x(t,x_{0})\equiv 0$. We therefore only need to show (13) for $x_{0}\ne 0$. Fix $x_{0}\ne 0$, let $x(t,x_{0})=x(t)$ is a solution of stochastic switched nonlinear system (1). By condition (10), we have $\ln [b_{i}|x|^{p}]\le \ln [V_{i}(t,x)]$.

According to $It\hat{o}$ formula, we obtain

$$\begin{aligned} \begin{aligned} d[\ln [V_{i}(t,x)]]&=\frac{1}{V_{i}(t,x)}\left[ \mathcal {L}V_{i}(t,x)-\frac{1}{2V_{i}(t,x)}\left( \frac{\partial V_{i}(t,x) }{\partial x}g_{i}(t,x)\right) ^{2}dt +\frac{\partial V_{i}(t,x)}{\partial x}g_{i}(t,x)dw(t)\right] . \end{aligned} \end{aligned}$$

where

$$\begin{aligned} \mathcal {L}V_{i}(t,x)=\frac{\partial V_{i}(t,x)}{\partial t}+\frac{\partial V_{i}(t,x)}{\partial x }f_{i}(t,x)+\frac{1}{2}tr\left[ g_{i}^{T}(t,x)\frac{\partial ^{2}V_{i}(t,x)}{\partial x ^{2}}g_{i}(t,x)\right] . \end{aligned}$$

We can give switching signal $$\sigma (t)$$ and instant *t* for arbitrary, and assume that $$t_{k}$$ is the last switching instant before *t*, i.e. there is no switching on the interval $$[t_{k},t)$$.

By $It\hat{o}$ formula, we have

$$\begin{aligned} \begin{aligned} \ln V_{\sigma (t_{k})}(t,x(t)) &=\ln V_{\sigma (t_{k})}(t_{k},x(t_{k}))+\int _{t_{k}}^{t} \mathcal {L}\ln V_{\sigma (t_{k})}(s,x(s))ds \\&+\int _{t_{k}}^{t} \frac{1}{V_{\sigma (t_{k})}(s,x(s))}\frac{\partial V_{\sigma (t_{k})}(s,x(s))}{\partial x}g_{\sigma (t_{k})}(s,x(s))dw(s) \\ &\le\ln [\eta V_{\sigma (t_{k-1})}(t_{k},x(t_{k}))]+\int _{t_{k}}^{t} \mathcal {L}\ln V_{\sigma (t_{k})}(s,x(s))ds \\&+\int _{t_{k}}^{t} \frac{1}{V_{\sigma (t_{k})}(s,x(s))}\frac{\partial V_{\sigma (t_{k})}(s,x(s))}{\partial x}g_{\sigma (t_{k})}(s,x(s))dw(s) \\ &=\ln [\eta V_{\sigma (t_{k-1})}(t_{k-1},x(t_{k-1}))]+\int _{t_{k-1}}^{t} \mathcal {L}\ln V_{\sigma (s)}(s,x(s))ds \\&+\int _{t_{k-1}}^{t} \frac{1}{V_{\sigma (s)}(s,x(s))}\frac{\partial V_{\sigma (s)}(s,x(s))}{\partial x}g_{\sigma (s)}(s,x(s))dw(s) \\ &\le\ln [\eta ^{k}V_{\sigma (t_{0})}(t_{0},x(t_{0}))]+\int _{t_{0}}^{t} \mathcal {L}\ln V_{\sigma (s)}(s,x(s))ds \\&+\int _{t_{0}}^{t} \frac{1}{V_{\sigma (s)}(s,x(s))}\frac{\partial V_{\sigma (s)}(s,x(s))}{\partial x}g_{\sigma (s)}(s,x(s))dw(s) \\ \end{aligned} \end{aligned}$$

Let $t_{0}=0$, we obtain

14 $$\begin{aligned} \begin{aligned} \ln V_{\sigma (t_{k})}(t,x(t)) &\le\ln [\eta ^{k} V_{\sigma (t_{0})}(0,x_{0})]+\int _{0}^{t}\frac{\mathcal {L}V_{\sigma (s) }(s,x(s))}{V_{\sigma (s) }(s,x(s))}ds \\&-\frac{1}{2}\int _{0}^{t}\frac{1}{V_{\sigma (s)}^{2}(s,x(s))}|\frac{\partial V_{\sigma (s)}(s,x(s))}{\partial x}g_{\sigma (s)}(s,x(s))|^{2}ds+M(t) \end{aligned} \end{aligned}$$

where $M(t)=\int _{0}^{t}\frac{1}{V_{\sigma (s)}(s,x(s))}\frac{\partial V_{\sigma (s)}(s,x(s))}{\partial x}g_{\sigma (s)}(s,x(s))dw(s)$ is a continuous local martingale with initial value $M(0)=0$.

Substituting (11) into (14) results in

$$\begin{aligned} \begin{aligned} \ln V_{\sigma (t_{k})}(t,x(t)) &\le\ln [\eta ^{k} V_{\sigma (t_{0})}(0,x_{0})]+\int _{0}^{t}\gamma \psi _{1}(s)ds -\frac{1}{2}\int _{0}^{t}\frac{1}{V_{\sigma (s)}^{2}(s,x(s))}|\frac{\partial V_{\sigma (s)}(s,x(s))}{\partial x}g_{\sigma (s)}(s,x(s))|^{2}ds+M(t) \\ &=\ln [\eta ^{k}V_{\sigma (t_{0})}(0,x_{0})]+\gamma \ln \psi (t)-\frac{1}{2}\int _{0}^{t}\frac{1}{V_{\sigma (s)}^{2}(s,x(s))}|\frac{\partial V_{\sigma (s)}(s,x(s))}{\partial x}g_{\sigma (s)}(s,x(s))|^{2}ds+M(t) \end{aligned} \end{aligned}$$

Therefore,

15 $$\begin{aligned} \begin{aligned} \ln |b_{\sigma (t_{k})}|x|^{p}|&\le\ln [\eta ^{k} V_{\sigma (t_{0})}(0,x_{0})]+\gamma \ln \psi (t) \\&-\frac{1}{2}\int _{0}^{t}\frac{1}{V_{\sigma (s)}^{2}(s,x(s))}|\frac{\partial V_{\sigma (s)}(s,x(s))}{\partial x}g_{\sigma (s)}(s,x(s))|^{2}ds+M(t) \end{aligned} \end{aligned}$$

Let $z(t)=\frac{1}{V_{\sigma (t)}(t,x(t))}\frac{\partial V_{\sigma (t)}(t,x(t))}{\partial x}g_{\sigma (t)}(t,x(t))$, by Lemma 6, $\forall n\in N, \forall \delta >0$, we have

$$\begin{aligned} P\left( \sup _{0\le t \le n+1}\left[ \frac{|\ln \psi (t)|}{t}M(t)-\frac{\delta }{2}\frac{|\ln \psi (t)|^{2}}{t^{2}}\int _{0}^{t}|z(s)|^{2}ds\right] \ge \frac{2 \ln n}{\delta }\right) \le \frac{1}{n^{2}}. \end{aligned}$$

By Lemma 7, when $n\rightarrow \infty ,\ n\le t \le n+1$, we obtain

$$\begin{aligned} \frac{|\ln \psi (t)|}{t}M(t)\le \frac{\delta }{2}\frac{|\ln \psi (t)|^{2}}{t^{2}}\int _{0}^{t}|z(s)|^{2}ds+\frac{2 \ln t}{\delta }, \quad a.s. \end{aligned}$$

That is

16 $$\begin{aligned} M(t)\le \delta \frac{|\ln \psi (t)|}{2t}\int _{0}^{t}|z(s)|^{2}ds+\frac{2t \ln t}{\delta |\ln \psi (t)|}, \quad a.s. \end{aligned}$$

Substituting (16) into (15) results in

$$\begin{aligned} \begin{aligned} \ln (b_{\sigma (t_{k})}|x|^{p})&\le\ln [\eta ^{k}V_{\sigma (t_{0})}(0,x_{0})]+\gamma \ln \psi (t)-\left( \frac{1}{2}-\frac{\delta |\ln \psi (t)|}{2t}\right) \int _{0}^{t}|z(s)|^{2}ds+\frac{2t \ln t}{\delta |\ln \psi (t)|} \\ &\le\ln [\eta ^{k}V_{\sigma (t_{0})}(0,x_{0})]+\gamma \ln \psi (t)-\left( \frac{1}{2}-\frac{\delta |\ln \psi (t)|}{2t}\right) t\max _{0\le s \le t}|z(s)|^{2}+\frac{2t \ln t}{\delta |\ln \psi (t)|} \\ &=\ln [\eta ^{k}V_{\sigma (t_{0})}(0,x_{0})]+\gamma \ln \psi (t)-\frac{t}{2}\max _{0\le s \le t}|z(s)|^{2}+\frac{\delta }{2}\max _{0\le s \le t}|z(s)|^{2} |\ln \psi (t)| +\frac{2t \ln t}{\delta |\ln \psi (t)|} \end{aligned} \end{aligned}$$

Then

$$\begin{aligned} \begin{aligned} \frac{\ln (b_{\sigma (t_{k})}|x|^{p})}{|\ln \psi (t)|}&\le \frac{\ln [\eta ^{k}V_{\sigma (t_{0})}(0,x_{0})]}{|\ln \psi (t)|}-\gamma -\frac{1}{2}\frac{t}{|\ln \psi (t)|}\max _{0\le s \le t}|z(s)|^{2}+\frac{\delta }{2}\max _{0\le s \le t}|z(s)|^{2} +\frac{2t \ln t}{\delta |\ln \psi (t)|^{2}} \end{aligned} \end{aligned}$$

Let $t \rightarrow \infty$, $\delta \rightarrow 0$, respectively. We have

$$\begin{aligned} \limsup _{t\rightarrow \infty }\frac{\ln (b_{\sigma (t_{k})}|x|^{p})}{|\ln \psi (t)|}\le -\gamma , \quad a.s. \end{aligned}$$

That is,

$$\begin{aligned} \limsup _{t\rightarrow \infty }\frac{\ln |x(t,x_{0})|}{|\ln \psi (t)|}\le -\frac{\gamma }{p} , \quad a.s. \end{aligned}$$

Thus, the system (1) is almost surely $\psi ^{\gamma }$ stable. $\square$

### *Remark 2*

Compared to the Theorem 3.3 and 6.2 given in Mao (1997), Theorem 1 and 2 extend the stability results from stochastic nonlinear systems to stochastic switched nonlinear systems. The systems given in Mao (1997) can be thought as a special case of system (1). Moreover, in Mao (1994, 1997) Filipovic (2009), Cong et al. (2011) the authors studied exponential stability, in Theorem 1 and 2, we investigated $\psi ^{\gamma }$ stability, the concept of $\psi ^{\gamma }$ stability contains exponential stability and polynomial stability, it has a wide applicability. In addition, for deterministic case, the researchers considered ordinary differential equations, in this paper, we considered stochastic differential equations.

## Examples

In this section, a numerical example is given to illustrate the effectiveness of the main results established in “Main results” section as follows.

Consider the stochastic switched nonlinear system (1). where $$\sigma (t): [t_{0},\infty )\rightarrow \{1,2\}$$ is the switching signal. Let $$\{t_{1}<t_{2}<\cdots<t_{k}<\cdots \}$$ be a switching sequence and the $$i_{k}$$th subsystem is active at time interval $$[t_{k},t_{k+1})$$, where $$t_{k}$$ is the switching instant, $$k=0,1,2,\cdots$$ and $$i_{k}\in \{1,2\}$$.

We choose $t_{k+1}-t_{k}=0.6s$, $V_{1}(t,x)=V_{2}(t,x)=x^{2}$, $\psi (t)=\frac{e^{-t}}{1+t},\ t\ge 0$, then, $\psi (0)=1, \psi (t)\rightarrow 0$ as $t\rightarrow \infty$, $\psi '(t)=-\frac{2+t}{(1+t)^{2}}e^{-t}$, $\psi _{1}(t)=\frac{\psi '(t)}{\psi (t)}=-(1+\frac{1}{1+t})$, $-2\le \psi _{1}(t)\le -1$.

When $\sigma (t)=1$, we choose $f_{1}(t,x)=-3x$ and $g_{1}(t,x)=\sqrt{2}x$ for the first subsystem; when $\sigma (t)=2$, we choose $f_{2}(t,x)=-2x-sgn x$ and $g_{2}(t,x)=\sqrt{2|x|}$ for the second subsystem.

For the first subsystem, If $x\ne 0$, we have

$$\begin{aligned} \begin{aligned} \mathcal {L}V_{1}(t,x)&=2x\cdot (-3x)+\frac{1}{2}\cdot 2\cdot 2x^{2}=-4x^{2} \\ &=-4V_{1}(t,x)\le 2\psi _{1}(t)V_{1}(t,x) \end{aligned} \end{aligned}$$

If $$x=0$$ then, $$V_{1}(t,x)=0, \ \mathcal {L}V_{1}(t,x)=0$$.

For the second subsystem, If $x\ne 0$, we have

$$\begin{aligned} \begin{aligned} \mathcal {L}V_{2}(t,x)&=2x\cdot (-2x-sgn x)+\frac{1}{2}\cdot 2\cdot 2\cdot |x|=-4x^{2} \\ &=-4V_{2}(t,x)\le 2\psi _{1}(t)V_{2}(t,x) \end{aligned} \end{aligned}$$

If $$x=0$$ then, $$V_{2}(t,x)=0, \ \mathcal {L}V_{2}(t,x)=0$$.

**Fig. 1:**
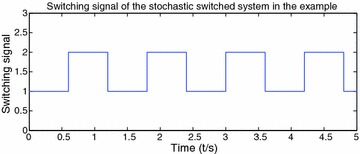
Switching signal of the stochastic switched nonlinear systems

**Fig. 2:**
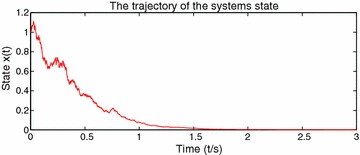
The trajectory of the stochastic switched nonlinear systems state

By Theorem 1, we can choose $p=2,\ \gamma =2$, then $\mathcal {L}V_{i}(t,x)\le \gamma \psi _{1}(t)V_{i}(t,x)$, $i=1,2$, which means that the conditions of Theorem 1 are satisfied. So the stochastic switched nonlinear systems are p-th moment $\psi ^{\gamma }$ stable. The switching signal and the state trajectory are presented in Figs. 1 and 2 respectively.

### *Remark 3*

In this example, a stochastic switched nonlinear system is constructed to show the efficiency of the main results. Figure 1 describes switching signal changes over the time. Figure 2 depicts state trajectory changes over the time and shows the system is p-th moment $\psi ^{\gamma }$ stability.

## Conclusions

In this paper, p-th moment and almost sure $\psi ^{\gamma }$ stability have been investigated for a class of stochastic switched nonlinear systems. Some sufficient conditions have been derived to check the stability criteria by using multiple Lyapunov functions approach. A numerical example is provided to verify the effectiveness of the main results. Our future research will be focus on $\psi ^{\gamma }$ stability of stochastic switched nonlinear systems with delay and $\psi ^{\gamma }$ stability of neutral stochastic switched nonlinear systems with delay.
